# Hypoglycemia during Short-Term Intensive Insulin Therapy and Its Association with Long-Term Glycemic Remission in Patients with Newly Diagnosed Type 2 Diabetes

**DOI:** 10.1155/2020/4097469

**Published:** 2020-02-18

**Authors:** Lijuan Xu, Pengyuan Zhang, Zhimin Huang, Liangying Zhong, Hai Li, Liehua Liu, Juan Liu, Haipeng Xiao, Yanbing Li

**Affiliations:** ^1^Department of Endocrinology, The First Affiliated Hospital, Sun Yat-Sen University, 58th of Zhongshan Er Road, Guangzhou, China 510080; ^2^Department of Laboratory Medicine, The First Affiliated Hospital, Sun Yat-Sen University, 58th of Zhongshan Er Road, Guangzhou, China 510080

## Abstract

**Background:**

Short-term intensive insulin therapy induces long-term glycemic remission in half of patients with newly diagnosed type 2 diabetes. The concomitant hypoglycemia needs further analysis.

**Methods:**

We collected data from three randomized trials conducted with the same inclusion and exclusion criteria at our institution from 2002 to 2015. Continuous subcutaneous insulin infusion (CSII) was provided to achieve the glycemic goals within a week and then maintained for 14 days. Hypoglycemia episodes during short-term treatment and the one-year drug-free glycemic remission were observed.

**Results:**

A total of 244 patients were included. The per day episode of mild hypoglycemia (3.0-3.9 mmol/L) was higher in the remission group than in the nonremission group (0.26 ± 0.20 vs. 0.18 ± 0.21, *P* = 0.005). However, a moderate hypoglycemia episode (<3.0 mmol/L) per day was insignificantly lower in the remission group (0.02 ± 0.04 vs. 0.03 ± 0.04, *P* = 0.005). However, a moderate hypoglycemia episode (<3.0 mmol/L) per day was insignificantly lower in the remission group (0.02 ± 0.04 vs. 0.03 ± 0.04, *P* = 0.005). However, a moderate hypoglycemia episode (<3.0 mmol/L) per day was insignificantly lower in the remission group (0.02 ± 0.04 vs. 0.03 ± 0.04, *P* = 0.005). However, a moderate hypoglycemia episode (<3.0 mmol/L) per day was insignificantly lower in the remission group (0.02 ± 0.04 vs. 0.03 ± 0.04,

**Conclusions:**

Mild hypoglycemic episodes during the continuing insulin dose reduction period indicate a long-term drug-free euglycemic remission in patients with newly diagnosed type 2 diabetes. However, the insulin dosage should be reduced even more quickly in the future treatment to decrease the potential harms.

## 1. Introduction

Diabetes is characterized by a progressive deterioration of *β*-cell function and reduction in insulin sensitivity [1, 2]. Glucose toxicity is an undisputed perpetrator of *β*-cell dysfunction that not only directly blunts the islet cell response to glucose but also accelerates *β*-cell dedifferentiation and apoptosis [3–6]. Short-term continuous subcutaneous insulin infusion (CSII) therapy is reported to rapidly alleviate glucotoxicity and restore the acute insulin response (AIR). Around half of the patients with newly diagnosed type 2 diabetes responded to CSII therapy and attained a long-term drug-free glycemic remission in our series of studies [7–9]. However, its use to achieve tight glucose control may cause an increase in hypoglycemia [10–12].

Hypoglycemia is classified into three levels based on the standard for hypoglycemia suggested by the recent American Diabetes Association (ADA) guideline [13], i.e., hypoglycemia alert value (mild hypoglycemia), clinically significant hypoglycemia (moderate hypoglycemia), and severe hypoglycemia. Severe hypoglycemia and clinically significant hypoglycemia are associated with an increased risk of macrovascular events, microvascular disease, and all-cause death [14–18]. Mild hypoglycemia is traditionally thought to be harmful and should be avoided if possible. However, a growing body of evidence suggests that mild hypoglycemia is not associated with CVD or all-cause mortality [19, 20]. Besides, we noticed that during CSII therapy, after reaching the normal glucose standard, some patients showed repeated mild hypoglycemia episodes despite the fact that their insulin doses decreased sharply. Furthermore, these patients tended to have better long-term drug-free blood glucose control. Whether the onset of mild hypoglycemia marked a long-term glycemic remission was unclear. Whether mild hypoglycemia and moderate hypoglycemia had different influences on long-term glycemic remission was inclusive. In addition to insulin use, what was associated with hypoglycemia was worth discussing.

For the above reasons, we analyzed the features of hypoglycemic episodes during the 2 to 3 weeks of CSII treatment and examined the association of hypoglycemia and the long-term drug-free glycemic control in this study.

## 2. Subjects and Methods

### 2.1. Subjects

Data was collected from three randomized prospective controlled trials conducted in 2002~2015 with the same inclusion and exclusion criteria (NCT00147836, NCT00948324, and NCT01471808, ClinicalTrials.gov) [8, 9, 21]. These trials were designed to compare the glucose control of short-term CSII alone with other therapy strategies. Only patients receiving CSII therapy alone were included in the current study to avoid interference from combinations of hypoglycemic agents. All subjects signed informed consent. The study protocols conformed to the provisions of the Declaration of Helsinki and were approved by the local Ethics Committee. Patients, 25 to 70 years old, with newly diagnosed type 2 diabetes, a fasting plasma glucose (FPG) level of 7.0-16.7 mmol/L, and a body mass index (BMI) of 21-35 kg/m^2^ were enrolled in these trials. Patients with acute or chronic microvascular complications of diabetes, or who had cardiovascular events or cerebrovascular diseases within 6 months, or who concomitantly suffered from severe diseases, or who had any condition that would greatly influence glycemic levels were excluded. Any other conditions (such as known drug or alcohol abuse or a psychiatric disorder) which might prevent the patient from following the therapeutic advice were also excluded.

### 2.2. Research Design

As previously described in detail [8, 9, 21], after the general evaluations and laboratory tests at the baseline, CSII therapy was then provided using insulin Lispro (Humalog, Eli Lilly Inc., USA) or Aspart (NovoRapid, Novo Nordisk, Denmark) to reach the glycemic targets (FPG levels 4.4-6.0 mmol/L and 2 h postprandial glucose (PPG) levels 4.4-8.0 mmol/L) as quickly and steadily as possible. The initial dosage was 0.4-0.8 IU/kg/day based on the patient's glucose level and physical condition. Half of the dosage was provided as a continuous basal infusion and the other half as boluses divided equally into three premeal infusions. Capillary blood glucose levels were assessed 8 times per day (before and 2 h after each meal, at bedtime, and at 3 AM) throughout CSII therapy. Whenever patients felt uncomfortable, the capillary blood glucose was extra checked. After reaching the glycemic targets, CSII therapy was maintained for another 14 days and then the insulin pump was removed before dinner time. The levels of FPG, PPG, HbA1c, and lipid profiles were checked on the next morning after more than 12 hours of discontinuing CSII treatment. Patients were given advice on continuous lifestyle improvements and followed up every 3 months. The one-year drug-free glycemic remission was observed during the follow-up visit. When a hyperglycemic relapse occurred, subsequent therapies were suggested according to the individual's glucose levels.

Glycemic remission was defined as FPG levels < 7.0 mmol/L and 2 h PPG levels < 10.0 mmol/L based on daily living measurement and with no need for hypoglycemic agents for at least one year. If the glucose levels exceeded this range, hyperglycemic relapse was considered to have occurred. Patients whose glucose levels met the criteria for glycemic remission were classified as the remission group, and patients whose levels did not meet these criteria were classified as the nonremission group during the one-year follow-up visit [21]. We evaluated the episodes of hypoglycemia during the CSII therapy based on the daily capillary blood glucose levels. A mild hypoglycemic episode was considered when the nadir blood glucose level at the alert value of 3.0-3.9 mmol/L was detected. Moderate hypoglycemia, also called clinically significant hypoglycemia, was regarded as a glucose level < 3.0 mmol/L. Severe hypoglycemia was defined as severe cognitive impairment requiring assistance from another person for recovery.

### 2.3. Measurements

General characteristics and anthropometric indices were evaluated. In particular, daily capillary blood glucose levels and hypoglycemic episodes were recorded. Laboratory indicators, including FPG, 2 h PPG, glycated hemoglobin (HbA1c), lipid profiles, and high-sensitivity C-reactive protein (hsCRP) levels, were tested. *β*-Cell function was estimated by assessing the AIR before and after CSII therapy. Homeostasis model assessment of *β*-cell function (HOMA-B) and homeostasis model assessment of insulin resistance (HOMA-IR) were also conducted.

### 2.4. Statistical Methods

All data were analyzed using the SPSS 22.0 Statistics (IBM, USA). Normally distributed data were expressed as means ± standard deviations (SDs) and compared by a *t*-test. Nonnormally distributed variables were expressed as medians (interquartile ranges) and analyzed by the rank-sum test. Chi-squared tests were conducted to analyze frequencies. In this study, most variables were normally distributed data. Levels of hsCRP, AIR, HOMA-B, and HOMA-IR were nonnormally distributed variables. The remission rate and the smoking rate were compared by Chi-squared tests. The number of hypoglycemic episodes was evaluated by both *t*-test and rank-sum test. Logistic regression was established to identify the potential influence of hypoglycemia on glycemic remission. Logistic regression and principal component analyses were used to explore the risk factors of hypoglycemia. A Cox regression model was used to test the association between hypoglycemia and long-term euglycemic remission. The *P* value < 0.05 was considered as statistically significant.

## 3. Results

### 3.1. General Conditions in the Remission Group and the Nonremission Group

We collected data of 124 patients from the clinical trial NCT00147836, 102 patients from NCT00948324, and 33 patients from NCT01471808. These patients received short-term CSII treatment alone. Three patients with slight infection and 12 patients lost to follow-up were excluded. Ultimately, a total of 244 patients with newly diagnosed type 2 diabetes were included in this analysis. After 2-3 weeks of CSII therapy, 127 (52.05%) patients attained one-year glycemic remission.

During the CSII maintaining period, glucose was well controlled within the target range. The levels of FPG were 5.07 ± 0.48 mmol/L in the remission group and 5.28 ± 0.51 in the nonremission group (*P* < 0.05). Levels of PPG after breakfast, lunch, and dinner were 7.40 ± 1.24, 7.42 ± 1.18, and 7.85 ± 1.39 mmol/L in the nonremission group and 6.63 ± 1.05, 6.90 ± 0.97, and 7.23 ± 1.19 mmol/L in the remission group (*P* < 0.05). After 2-3 weeks of intensive insulin therapy, glucose and lipid profiles were considerably improved. HbA1c was reduced from 10.53 ± 2.35% at the baseline to 8.96 ± 1.83% after treatment (*P* < 0.001). FPG dropped from 12.48 ± 3.88 mmol/L at the baseline to 6.35 ± 1.31 after cessation of CSII overnight (*P* < 0.001). Similarly, PPG significantly decreased from 18.25 ± 6.01 to 8.28 ± 2.39 after discontinuing treatment overnight. Comparing the levels at the baseline and after treatment, those of TG, HDL-C, and LDL-C were 2.14 ± 1.60 vs. 1.39 ± 0.59, 1.13 ± 0.28 vs. 1.23 ± 0.28, and 3.71 ± 1.15 vs. 3.37 ± 1.03 mmol/L, respectively (*P* < 0.001). The level of hsCRP was also reduced after treatment compared with that at the baseline (1.30 (2.56) vs. 1.57 (2.22), *P* = 0.005).

Several differences were identified between the remission and nonremission groups. The proportion of men in the remission and nonremission groups was 58.23% and 41.78%. And the proportion of women was 40.70% and 59.30%. Men have a higher glycemic response rate than women (*P* = 0.011). Patients who attained glycemic remission were slightly younger than those who did not (48.06 ± 9.85 vs. 50.95 ± 11.70 years old, *P* = 0.037). Surprisingly, baseline AIR tended to be lower in the remission group than that in the nonremission group (-125.71 (263.04) vs. -99.36 (230.29)), although insignificantly (*P* = 0.589). After short-term CSII treatment, those who attained glycemic remission had more improved AIR (491.35 (801.89) vs. 370.22 (542.29), *P* = 0.028) and HOMA-IR (2.08 (2.04) vs. 2.48 (2.32), *P* = 0.038) than those who did not (Tables 1 and 2).

### 3.2. Differences in Insulin Doses and Glucose Profiles between the Remission Group and the Nonremission Group

**Figure 1:**
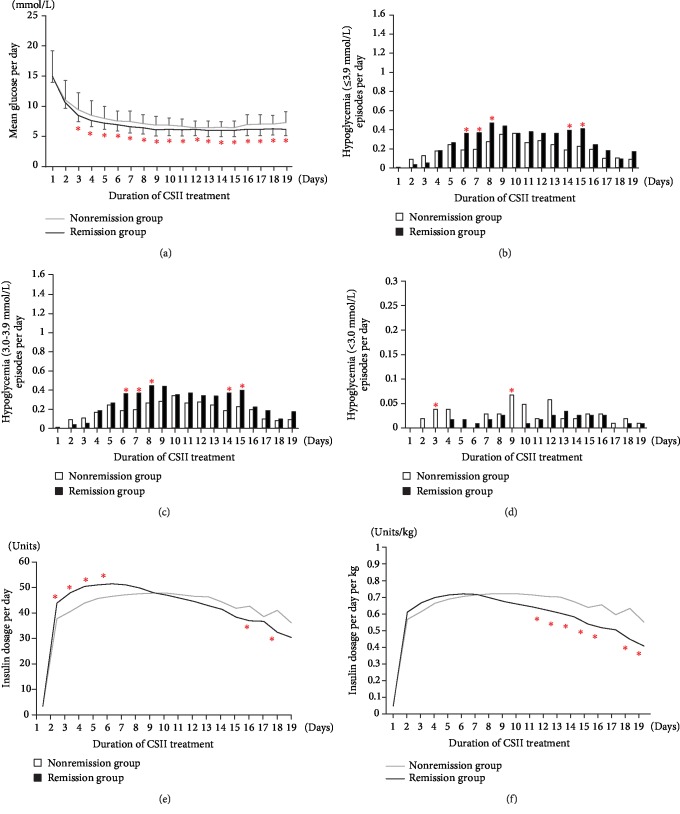
Glucose profiles (a), daily hypoglycemic episodes (b–d), and daily insulin doses (e, f) of patients during continuous subcutaneous insulin infusion (CSII) therapy in the remission group and the nonremission group. Compared with those in the nonremission group, patients in the remission group had a lower mean glucose, an increased mild hypoglycemia episode, a higher initial insulin dose, and a lower dose before removal of the insulin pump. Glucose profiles (a) and daily insulin doses (e, f) were compared by *t*-test. The number of hypoglycemic episodes (b–d) was calculated by rank-sum tests. ∗ means *P* < 0.05.

The initial insulin dose in the remission group was greater than that in the nonremission group (45.04 ± 15.45 vs. 39.44 ± 12.04 IU, *P* = 0.002), but when body weight was adjusted, the per kilogram dose became insignificantly higher in the remission group (0.64 ± 0.20 vs. 0.59 ± 0.16 IU/kg, *P* = 0.072). There were no differences in insulin dosage peak values between the two groups (51.35 ± 18.86 vs. 47.80 ± 15.86 IU, *P* = 0.153; 0.72 ± 0.23 vs. 0.73 ± 0.24 IU/kg, *P* = 0.964). However, the peak-reaching time of the insulin dosage was shorter in the remission group than in the nonremission group (6.85 ± 4.37 vs. 9.07 ± 4.76 days, *P* < 0.001). In addition, the insulin dosage before the cessation of CSII was much lower in the remission group than in the nonremission group (31.88 ± 20.84 vs. 41.10 ± 18.50 IU, *P* = 0.016; 0.45 ± 0.28 vs. 0.64 ± 0.30 IU/kg, *P* < 0.001). The average daily insulin dose was similar in the two groups during CSII treatment (40.42 ± 12.04 vs. 40.54 ± 10.72 IU, *P* = 0.937), but the average dose per kilogram body weight was lower in the remission group than in the nonremission group (0.56 ± 0.17 vs. 0.61 ± 0.16 IU/kg, *P* = 0.013) (Figure 1).

The time of glucose reaching targets was 1.81 ± 1.04 days in the remission group and 2.03 ± 1.04 days in the nonremission group (*P* = 0.091). Glucose decreased more rapidly and maintained at lower levels during CSII therapy in the remission group, even though the insulin dose was much lower in the remission group than in the nonremission group before CSII therapy stopped. After terminating the short-term CSII therapy overnight, significantly lower FPG and PPG levels were observed in the remission group than in the nonremission group (6.07 ± 1.27 vs. 6.66 ± 1.29 mmol/L for FPG; 7.70 ± 2.28 vs. 8.95 ± 2.43 mmol/L for PPG; *P* < 0.001) (Table 2 and Figure 1).

### 3.3. Hypoglycemic Episodes during CSII Therapy

Although the insulin dose was carefully titrated, patients experienced 0.21 ± 0.22 mild hypoglycemia and 0.02 ± 0.04 moderate hypoglycemia episodes per day during the 2~3 weeks of CSII therapy. Fifteen patients experienced more than one moderate hypoglycemia episode during the 2~3 weeks of intensive insulin therapy. No severe hypoglycemia requiring assistance from other individuals occurred; all patients rapidly recovered following calorie intake and insulin dose titration. No severe adverse events were reported.

Mild hypoglycemia episodes were the most before lunch and the least after supper (1.18 ± 1.57 vs. 0.20 ± 0.52, *P* < 0.001). Those in the morning, before bedtime, and at midnight were 0.57 ± 1.01, 0.72 ± 1.27, and 0.78 ± 1.38, respectively (*P* = 0.100). The occurrence of moderate hypoglycemia had the similar trend. The most and least moderate hypoglycemia episodes were 0.11 ± 0.35 before lunch and 0.01 ± 0.11 after supper (*P* < 0.001). Those in the morning, before bedtime, and at midnight were 0.04 ± 0.24, 0.08 ± 0.36, and 0.04 ± 0.21, respectively (*P* = 0.698).

### 3.4. The Occurrence of Hypoglycemia in Different Genders and Ages

During CSII treatment, there was no difference between men and women in either mild hypoglycemia (4.53 ± 3.98 vs. 3.76 ± 3.92, *P* = 0.166) or moderate hypoglycemia (0.40 ± 0.75 vs. 0.45 ± 0.80, *P* = 0.615). We divided the patients into the group of <45 years old, 45~55 years old, and >55 years old and observed the occurrence of hypoglycemia in different age groups. Mild hypoglycemia episodes were 4.14 ± 3.76, 4.70 ± 4.38, and 4.04 ± 3.84 in the three groups during the whole course of CSII treatment (*P* = 0.794). The total episodes of moderate hypoglycemia appeared higher in patients older than 55 years, but not statistically significant (0.37 ± 0.74, 0.33 ± 0.60, and 0.54 ± 0.89, *P* = 0.153).

### 3.5. Different Features of Hypoglycemia in the Remission Group and the Nonremission Group

Significantly more episodes of mild hypoglycemia per day were observed in the remission group than in the nonremission group (0.26 ± 0.20 vs. 0.18 ± 0.21, *P* = 0.005). However, the incidence of moderate hypoglycemia (<3.0 mmol/L) per day was insignificantly lower in the remission group (0.02 ± 0.04 vs. 0.03 ± 0.04, *P* = 0.221). During the 6-8^th^ day, the episode of mild hypoglycemia in the remission group was significantly higher in the remission group than that in the nonremission group (0.38 ± 0.44 vs. 0.20 ± 0.36, *P* = 0.001). Correspondingly, the mean insulin dose during these days tended to be higher in the remission group (50.67 ± 18.96 vs. 47.17 ± 14.99 IU/d, *P* = 0.154). However, on the 14-15^th^ day of insulin therapy, there was a second peak of mild hypoglycemic episodes in the remission group even though the insulin dose was decreased from the maximum to a much lower level (51.35 ± 18.86 vs. 38.28 ± 20.19 IU/d, *P* < 0.001), whereas the moderate hypoglycemia episodes did not increase simultaneously. During the whole insulin dose reduction stage, although insulin doses had decreased insignificantly lower in the remission group than in the nonremission group (37.93 ± 18.58 vs. 41.85 ± 15.87, *P* = 0.122), more mild hypoglycemic episodes per day were demonstrated (0.34 ± 0.32 vs. 0.22 ± 0.27, *P* = 0.006). When the rank-sum test of nonnormal distribution was used for the above indices, the same results were obtained (Table 2 and Figure 1).

### 3.6. Association between Mild Hypoglycemia during the CSII Therapy and the One-Year Euglycemic Remission

**Figure 2:**
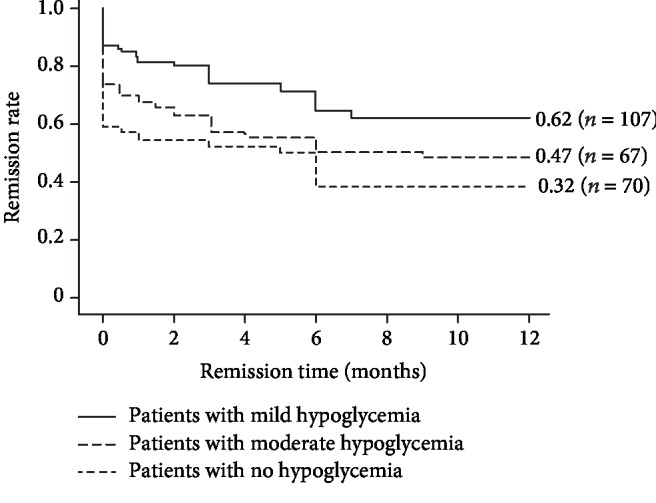
Distinct remission rates during the one-year follow-up in patients with only mild hypoglycemia episode, moderate hypoglycemia episode, or no hypoglycemic occurrence. The Cox regression model was used to test the association between hypoglycemia and long-term euglycemic remission. The remission rate was the highest in patients with mild hypoglycemia (62.04% vs. 47.06% vs. 31.82%, *P* < 0.05).

Based on the comparisons in the present study, gender, age, BMI, mild hypoglycemic episodes, average insulin dosage of CSII, posttreatment FPG, PPG, AIR levels, and HOMA-IR values significantly differed between the remission and the nonremission groups. Among the above parameters, only mild hypoglycemic episodes (OR = 2.18, 95% CI 1.02~4.70) and posttreatment FPG levels (OR = 0.64, 95% CI 0.46~0.90) were independently correlated with long-term glycemic remission according to the logistic regression analysis. When these continuous variables were stratified based on the characteristics of clinical indicators, the same association was demonstrated (Table 3). Subgroup analysis showed that mild hypoglycemic episodes during the insulin dosage reduction period instead of increase period were correlated with long-term glycemic remission. Survival curves revealed that patients who experienced only mild hypoglycemia had significantly higher remission rates than patients who either developed moderate hypoglycemia or who experienced no hypoglycemia during the 12-month follow-up (62.04% vs. 47.06% vs. 31.82%, *P* < 0.05, Figure 2).

### 3.7. Baseline Factors Related to Moderate Hypoglycemic Episodes

We compared baseline parameters between patients with moderate hypoglycemia and patients with only mild hypoglycemia and found that those who experienced moderate hypoglycemic episodes had higher baseline levels of HbA1c, FPG, PPG, TC, LDL-C, hsCRP, and initial insulin dosage. According to the principal component analysis and logistic regression analysis, patients with higher glucose levels (HbA1c, FPG, and PPG, OR = 2.60, 95% CI 1.75-3.85, *P* < 0.001) and those with higher cholesterol levels (TC and LDL-C, OR = 1.61, 95% CI 1.14-2.27, *P* = 0.007) at the baseline had an increased risk of moderate hypoglycemia.

## 4. Discussion

In the present study, we analyzed the influence of mild hypoglycemia with nadir glucose levels of 3.0-3.9 mmol/L and moderate hypoglycemia (<3.0 mmol/L) during the short-term CSII treatment on the long-term glucose remission. Interestingly, mild hypoglycemic episodes especially during the continuing insulin dose decrease stage were positively correlated with long-term glycemic remission. However, moderate hypoglycemic episodes were insignificantly higher in the nonremission group.

During CSII therapy, there were two peaks of mild hypoglycemia episodes in the remission group. The first peak appeared along with the increase of insulin doses. The second peak occurred on the 15^th^ day of CSII therapy, when insulin doses tended to be lower in the remission group. It suggests a more improved insulin resistance, and thus, the lower dosage of insulin has a larger effect on glucose control and led to hypoglycemia. What is more, the moderate hypoglycemia did not increase simultaneously, indicating a more complete improvement of endogenous islet function. After CSII treatment cessation, both AIR and HOMA-IR demonstrated more significant improvement in the remission group, which also supported our point of view.

This result is partly coincident with another study, in which responders have lower glycemia and less hypoglycemia from the third week onwards of CSII therapy [22], suggesting that 2 weeks of therapy may be needed to improve endogenous islet function when intensive insulin therapy is conducted. However, further studies are necessary to determine the exactly optimal duration of CSII treatment by evaluating the index of *β*-cell function and insulin resistance at different treatment courses.

Whereas in our previous study, we did not found differences of hypoglycemia between patients in the remission group and those in the nonremission group [23]. The reason was that in our previous studies, hypoglycemia was not analyzed by subgroups. When hypoglycemia was divided into minor hypoglycemia (3.0-3.9 mmol/L) and moderate hypoglycemia (<3.0 mmol/L), the results showed a significant difference in mild hypoglycemia between the two groups.

Interestingly, more and more studies showed that mild hypoglycemia was not associated with CVD events and all-cause death [19, 20]. In the Action in Diabetes and Vascular Disease: Preterax and Diamicron Modified Release Controlled Evaluation (ADVANCE) study, risks of incident macrovascular outcomes and deaths in those experiencing minor hypoglycemia were even found much lower compared to those not reporting minor hypoglycemia [19]. In another study, recurrent mild hypoglycemia had been reported to precondition and protect the brain from neuronal damage and cognitive deficits induced by severe hypoglycemia in rats [24]. Notwithstanding, most studies have shown that mild hypoglycemia might be harmful [25, 26]. Although in our study mild hypoglycemia episodes during the continuing insulin dose decrease stage were associated with better long-term glucose control, we must caution that the positive correlation between mild hypoglycemia and glycemic remission is only applicable to patients with newly diagnosed type 2 diabetes who do not present with acute or chronic complications or severe concomitant diseases.

Therefore, we need to reduce the insulin dosage even more quickly when blood glucose reaches the target to decrease the occurrence of hypoglycemia as much as possible in the future treatment. Furthermore, moderate hypoglycemic episodes did not contribute to glycemic remission. In addition to the insulin dose, moderate hypoglycemia was associated with baseline glucose levels and lipid profiles. Patients who display higher glucose and cholesterol levels before intensive insulin therapy are at an increased risk of developing moderate hypoglycemia, and their insulin dosage should be adjusted particularly carefully.

In this study, we find that hypoglycemia is more frequent before lunch instead of at midnight. The reasons may be as follows. First, it is related to Chinese breakfast habit. A Chinese breakfast mainly consists of easily absorbed carbohydrates, such as porridge and noodles. These foods cause blood glucose to rise and fall rapidly and thus leading to a decreased level of glucose before the next meal. Second, monitoring midnight glucose only at three o'clock may miss some unrecognized hypoglycemia detection and cause certain bias.

To the best of our knowledge, this study is the first to investigate the impact of mild hypoglycemia (3.0-3.9 mmol/L) and moderate hypoglycemia (<3.0 mmol/L), respectively, during short-term CSII therapy on long-term glycemic remission. However, several limitations should be considered in the interpretation of our results. The current study is a post hoc analysis of three prospective clinical trials. Therefore, we cannot eliminate selection bias and subtle differences among the trials even though these studies were conducted with similar inclusive criteria, exclusive criteria, and insulin dose titration procedure. Another limitation is that although we monitored the glucose levels at 3am, a continuous glucose monitoring system (CGMS) was not provided to most patients, so asymptomatic nocturnal hypoglycemia might have been partly missed in our study. In addition, changes in glycosylated albumin before and after short-term CSII treatment, which we unfortunately did not check in this study, may better reflect the changes of glucose than HbA1c, considering that glycosylated albumin represents the past 2-3 weeks of glucose.

In conclusion, half of patients with newly diagnosed type 2 diabetes can respond to the short-term intensive insulin treatment and maintain a long-term drug-free glycemic remission. The incidence of mild hypoglycemia is higher, and moderate hypoglycemia tends to be lower in the remission group. Mild hypoglycemia that occurs when the insulin dose continues to decrease indicates a long-term drug-free euglycemic remission. However, in the future treatment with CSII, insulin dosage should be reduced even more quickly when blood glucose reaches the target to decrease the potential harms caused by hypoglycemia.

## Figures and Tables

**Table 1:** Differences between the remission group and the nonremission group at the baseline.

	Remission group	Nonremission group	*t* or *Z* value	*P* value
Number	127	117	—	—
Gender (F/M)	35/92	51/66	2.613	0.011^∗^
Age (years)	48.06 ± 9.85	50.95 ± 11.70	-2.096	0.037^∗^
Smoking habit	25.20%	20.51%	0.867	0.386
BMI (kg/m^2^)	25.53 ± 3.52	24.66 ± 3.20	2.007	0.046^∗^
HbA1c (%) (mmol/mol)	10.54 ± 2.38 91.00 ± 14.50	10.52 ± 2.31 91.00 ± 13.50	0.085	0.932
FPG (mmol/L)	12.00 ± 3.52	12.95 ± 4.17	-1.927	0.055
PPG (mmol/L)	18.52 ± 6.46	17.34 ± 5.49	1.503	0.134
Triglyceride (mmol/L)	2.25 ± 1.84	2.20 ± 2.68	0.179	0.858
Cholesterol (mmol/L)	5.72 ± 1.20	5.93 ± 1.20	-1.371	0.172
HDL-C (mmol/L)	1.09 ± 0.29	1.17 ± 0.28	-2.147	0.033^∗^
LDL-C (mmol/L)	3.66 ± 1.19	3.75 ± 1.13	-0.602	0.548
hsCRP (mg/L)	1.70 (2.59)	1.33 (2.17)	0.982	0.326
AIR (pmol/L·10 min)	-125.71 (263.04)	-99.36 (230.29)	0.541	0.589
HOMA-B	24.47 (31.99)	23.85 (24.02)	1.206	0.228
HOMA-IR	4.20 (4.30)	4.88 (4.95)	0.893	0.373

∗ means *P* < 0.05. BMI: body mass index; HbA1c: glycated hemoglobin; FPG: fasting plasma glucose; PPG: postprandial plasma glucose; HDL-C: high-density lipoprotein cholesterol; LDL-C: low-density lipoprotein cholesterol; hsCRP: high-sensitivity C-reactive protein; AIR: acute insulin response; HOMA-B: homeostasis model assessment of *β*-cell function; HOMA-IR: homeostasis model assessment of insulin resistance; AIR: acute insulin response.

**Table 2:** Differences between the remission group and the nonremission group during and after CSII cessation.

	Remission group	Nonremission group	*t* or *Z* value	*P* value
*After CSII treatment*				
BMI decrease (kg/m^2^)	0.28 ± 0.80	0.18 ± 1.02	0.769	0.443
HbA1c (%) (mmol/mol)	8.91 ± 1.86 74.00 ± 8.50	9.03 ± 1.80 75.00 ± 8.00	-0.494	0.622
FPG (mmol/L)	6.07 ± 1.27	6.66 ± 1.29	-3.626	<0.001^∗^
PPG (mmol/L)	7.70 ± 2.28	8.95 ± 2.43	-3.861	<0.001^∗^
Triglyceride (mmol/L)	1.33 ± 0.62	1.46 ± 0.55	-1.704	0.090
Cholesterol (mmol/L)	5.24 ± 1.09	5.37 ± 1.02	-0.943	0.347
HDL-C (mmol/L)	1.22 ± 0.31	1.25 ± 0.34	-0.639	0.524
LDL-C (mmol/L)	3.40 ± 1.06	3.34 ± 0.99	0.436	0.663
hsCRP (mg/L)	1.37 (3.01)	1.28 (1.71)	0.256	0.798
AIR (pmol/L∗10 min)	491.35 (801.89)	370.22 (542.29)	2.191	0.028^∗^
HOMA-B	65.00 (73.70)	65.05 (66.86)	1.250	0.211
HOMA-IR	2.08 (2.04)	2.48 (2.32)	2.074	0.038^∗^
*During CSII treatment*				
Mean insulin dose (IU/kg)	0.56 ± 0.17	0.61 ± 0.16	-2.499	0.013^∗^
Mean insulin dose during insulin increase stage (IU/kg)	0.74 ± 0.20	0.73 ± 0.18	0.423	0.673
Mean insulin dose during insulin decrease stage (IU/kg)	0.60 ± 0.23	0.65 ± 0.22	-1.466	0.144
Mild hypoglycemic episodes per day (3.0~3.9 mmol/L)	0.26 (0.16)	0.11 (0.16)	3.518	<0.001^∗^
	0.26 ± 0.20	0.18 ± 0.21	2.824	0.005^∗^
Moderate hypoglycemic episodes per day (<3.0 mmol/L)	0 (0.05)	0 (0.05)	1.097	0.273
	0.02 ± 0.04	0.03 ± 0.04	-1.228	0.221
Mild hypoglycemia episodes per day during insulin increase stage	0 (0.08)	0 (0.07)	0.094	0.925
	0.06 ± 0.12	0.06 ± 0.13	-1.290	0.118
Mild hypoglycemia episodes per day during insulin decrease stage	0.29 (0.46)	0.13 (0.30)	2.974	0.003^∗^
	0.34 ± 0.32	0.22 ± 0.27	2.820	0.006^∗^

∗ means *P* < 0.05. BMI: body mass index; HbA1c: glycated hemoglobin; FPG: fasting plasma glucose; PPG: postprandial plasma glucose; HDL-C: high-density lipoprotein cholesterol; LDL-C: low-density lipoprotein cholesterol; hsCRP: high-sensitivity C-reactive protein; AIR: acute insulin response; HOMA-B: homeostasis model assessment of *β-*cell function; HOMA-IR: homeostasis model assessment of insulin resistance.

**Table 3:** Influence factors of one-year euglycemia remission.

	Odds ratio	95% CI for OR	*P* value
(A) Overall analysis			
Gender	1.342	0.632~2.850	0.444
Age	0.992	0.961~1.024	0.616
BMI at baseline	1.055	0.949~1.172	0.323
HDL-C at baseline	0.446	0.123~1.613	0.218
Mild hypoglycemia (3.0~3.9 mmol/L)	2.184	1.015~4.699	0.046^∗^
Mean insulin dose (IU/day)	0.992	0.962~1.023	0.605
FPG after treatment	0.641	0.456~0.902	0.011^∗^
PPG after treatment	0.912	0.787~1.056	0.217
AIR after treatment	1.000	1.000~1.001	0.849
HOMA-IR after treatment	0.976	0.807~1.181	0.806
(B) Stratified analysis			
Gender	1.439	0.673~3.077	0.348
Men			
Women			
Age (years)	0.901	0.588~1.380	0.633
<45			
45-55			
>55			
BMI at baseline (kg/m^2^)	1.062	0.639~1.767	0.816
<24			
24-28			
>28			
HDL-C at baseline (mmol/L)	0.623	0.305~1.269	0.192
<1.0			
≥1.0			
Total episodes of mild hypoglycemia	1.593	1.153~2.201	0.005^∗^
None			
1-2			
3-5			
>5			
Mean insulin dose (IU/day)	0.903	0.509~1.600	0.726
<30			
30-40			
>40			
FPG after treatment (mmol/L)	0.661	0.442~0.989	0.044^∗^
<5.0			
5.0-6.0			
6.1-7.0			
>7.0			
PPG after treatment (mmol/L)	0.697	0.467~1.039	0.076
<6.0			
6.0-8.0			
8.1-10.0			
>10.0			
AIR after treatment (pmol/L·10 min)	1.333	0.718~2.472	0.362
<0			
0-430			
>430			
HOMA-IR after treatment	1.271	0.666~2.426	0.466
<2.25			
≥2.25			

∗ means *P* < 0.05. BMI: body mass index; FPG: fasting plasma glucose; PPG: postprandial plasma glucose; HDL-C: high-density lipoprotein cholesterol; AIR: acute insulin response; HOMA-IR: homeostasis model assessment of insulin resistance. The median levels of AIR and HOMA-IR were 430 pmol/L·10 min and 2.25, respectively. Age, BMI, HDL-C, total episodes of mild hypoglycemia, mean insulin dose, FPG, and PPG were stratified according to the characteristics of clinical indicators. AIR and HOMA-IR were stratified based on the median levels.

## Data Availability

The data used to support the findings of this study are available from the corresponding author upon request.
